# Biomarkers in Rheumatoid Arthritis: From Traditional Serology to Precision Medicine Integration

**DOI:** 10.3390/diagnostics16020330

**Published:** 2026-01-20

**Authors:** Muhammad Soyfoo, Julie Sarrand

**Affiliations:** Department of Rheumatology, Hôpital Universitaire de Bruxelles, Université libre de Bruxelles, 1070 Brussels, Belgium; julie.sarrand@ulb.be

**Keywords:** rheumatoid arthritis, biomarkers, anti-citrullinated protein antibodies, calprotectin, 14-3-3η, multi-biomarker disease activity, ultrasound, MRI, precision medicine, treat-to-target

## Abstract

The biomarker landscape in rheumatoid arthritis (RA) is evolving from reliance on traditional markers toward integrated, multimodal strategies enabling precision medicine approaches. To critically evaluate emerging biomarkers across serological, cellular, genetic, imaging, and multi-omic domains, distinguishing those approaching clinical readiness from those requiring further development. In this study, a narrative review of the literature published between 2000 and 2024 relevant to clinical decision-making in RA was conducted. Among novel serological markers, 14-3-3η protein and anti-carbamylated protein antibodies show the strongest validation for seronegative disease and prognostic stratification. Calprotectin demonstrates utility for disease activity monitoring and de-escalation decisions. Multi-biomarker disease activity scores provide an objective assessment but lack outcome trial validation. Musculoskeletal ultrasound offers accessible imaging biomarker capability, while MRI bone marrow edema remains the strongest structural progression predictor. Synovial tissue pathotyping has demonstrated proof-of-concept for treatment stratification. Genetic, epigenetic, and metabolomic approaches remain investigational. Key clinical implications include using 14-3-3η and calprotectin to inform seronegative diagnosis and de-escalation decisions, integrating ultrasound for remission verification, and recognizing that emerging biomarkers for extra-articular complications, including cardiovascular risk and venous thromboembolism, represent important unmet needs.

## 1. Introduction

The biomarker landscape in rheumatoid arthritis (RA) stands at an inflection point [1]. For decades, clinical practice has relied on RF (rheumatoid factor) and ACPAs (anti-citrullinated protein antibodies) for diagnosis and CRP (C-reactive protein)/ESR (erythrocyte sedimentation rate) for disease activity monitoring—an approach that, while foundational, fails to address key clinical challenges [2]. The emerging paradigm shifts from single biomarkers to integrated multimodal strategies, from static diagnosis to dynamic risk stratification, and from population-based algorithms to precision medicine tailored to individual disease biology. This evolution is driven by recognition that RA encompasses heterogeneous disease subsets with distinct pathophysiological mechanisms, treatment responses, and outcomes [1,3].

**Figure 1:**
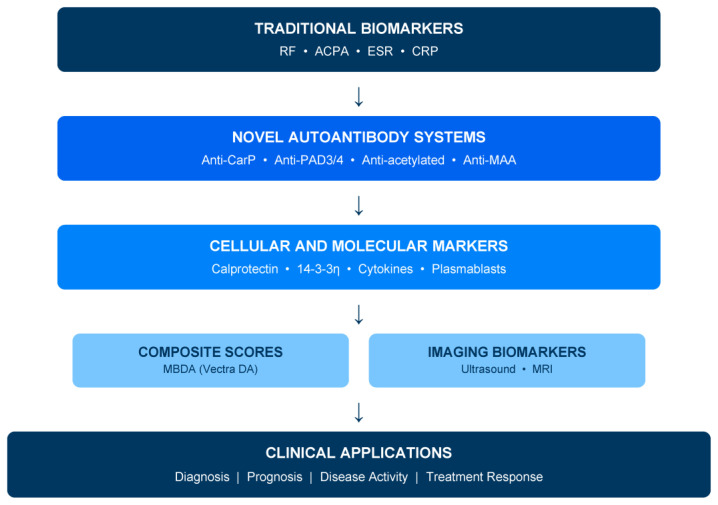
Biomarker hierarchy in rheumatoid arthritis: from traditional to emerging markers. This framework is intended to guide expectations regarding clinical readiness rather than mandate specific testing sequences. Tier placement reflects current validation status, commercial availability, and guideline incorporation. Movement between tiers requires demonstration of clinical utility, assay standardization, and cost-effectiveness. RF: rheumatoid factor; ACPA: anti-citrullinated protein antibodies; CRP: C-reactive protein; ESR: erythrocyte sedimentation rate; MBDA: multi-biomarker disease activity; Anti-CarP: anti-carbamylated protein antibodies; Anti-PAD: anti-peptidylarginine deiminase antibodies; Anti-MAA: anti-malondialdehyde–acetaldehyde antibodies.

Three clinical scenarios illustrate where novel biomarkers could transform practice. Firstly, the seronegative early arthritis patient presenting with inflammatory symptoms but negative RF and ACPAs poses a diagnostic challenge; novel autoantibodies may enable earlier, more confident diagnosis and timely treatment initiation [4]. Secondly, the patient in apparent clinical remission with low disease activity scores may harbor subclinical synovitis predicting structural progression or flare—biomarkers detecting this discordance could guide de-escalation decisions with greater confidence [5,6]. Thirdly, identifying patients at high risk for aggressive, erosive disease at presentation could justify early biologic therapy, while recognizing those likely to achieve sustained remission might support treatment tapering strategies (Figure 1) [7,8].

The concept of the ‘window of opportunity’ underscores that early aggressive treatment prevents irreversible joint damage [7,8]. This window extends beyond established disease to preclinical and at-risk phases, where autoantibodies and multi-omic signatures may identify individuals destined to develop RA years before symptom onset—a population where preventive intervention trials are underway [9,10]. Biomarkers enabling this preclinical identification represent a frontier of particular translational importance.

A critical distinction must frame this review: the gap between biomarker discovery and biomarkers ready for clinical practice. Many promising candidates demonstrate strong associations in research cohorts but lack the validation, standardization, and demonstrated clinical utility required for implementation [4,11]. Throughout this review, biomarkers are categorized along this discovery-to-practice continuum, distinguishing those approaching clinical readiness (calprotectin, 14-3-3η, MBDA, ultrasound) from those requiring further development (most genetic, epigenetic, and proteomic markers). This framework aims to provide clinicians with actionable guidance while acknowledging the evolving evidence base. In summary, this review addresses three transformative clinical scenarios where novel biomarkers hold particular promise: first, triaging seronegative early arthritis where traditional serology fails to confirm diagnosis; second, detecting subclinical inflammation in patients achieving apparent clinical remission to inform de-escalation decisions; and third, predicting flares or aggressive disease trajectory to guide treatment intensity.

## 2. Methods

This narrative review synthesizes literature from PubMed, EMBASE, and Cochrane databases (2000–2024), focusing on biomarkers with diagnostic, prognostic, or disease activity monitoring applications in RA. Search terms included combinations of “rheumatoid arthritis” with “biomarker”, “autoantibody”, “calprotectin”, “14-3-3η”, “MBDA”, “ultrasound”, “MRI”, and “precision medicine”. We prioritized meta-analyses, prospective cohort studies, and clinical trials where available. Studies were included if they evaluated biomarker performance in adult RA populations with clearly defined outcomes (diagnosis, prognosis, disease activity, or treatment response). Cross-sectional studies and case reports were generally excluded unless they provided unique mechanistic insights. Real-life cohort data were included when prospective validation studies were lacking. Key biomarkers were prioritized based on replication across independent cohorts, availability of commercial assays, and proximity to clinical implementation. No formal quality assessment tool was applied, given the narrative nature of this review; however, we explicitly distinguish between biomarkers with strong prospective validation versus those remaining at earlier discovery stages. Our central thesis is that no single biomarker will transform RA management; rather, clinically meaningful progress will come from rational integration of serological, inflammatory, and imaging markers applied longitudinally within a treat-to-target framework [12].

## 3. Traditional Biomarkers: Established Utility and Recognized Limitations

RF and ACPAs remain the serological foundation of RA diagnosis, incorporated into the 2010 ACR/EULAR classification criteria [2]. RF demonstrates sensitivity of 60–80% and specificity of 70–85%, while anti-CCP assays achieve comparable sensitivity (60–75%) with superior specificity (95–99%) [13]. Beyond binary positivity, quantitative aspects carry prognostic significance: high-titer RF (particularly IgA isotype) and high-titer ACPAs are associated with more aggressive disease phenotypes, extra-articular manifestations, and accelerated radiographic progression [3,14]. The magnitude of ACPA elevation correlates with erosive burden, with very high titers (>3 times the upper limit of normal) identifying patients at greatest structural damage risk. ACPAs may appear years before clinical onset, establishing their value in preclinical risk assessment—a finding with implications for emerging prevention trials in at-risk populations [9,10].

Acute phase reactants (CRP, ESR) remain integral to composite disease activity measures (DAS28, SDAI, CDAI) but reflect systemic inflammation rather than joint-specific pathology [1]. Despite decades of clinical use, these traditional biomarkers carry well-recognized limitations that drive the search for novel markers [3,4]:

Seronegativity: Approximately 20–30% of RA patients remain RF-negative and ACPA-negative, delaying diagnosis and potentially missing the therapeutic window of opportunity [4,13].

Clinical-serological discordance: A substantial subset exhibits active clinical disease despite normal CRP/ESR, while others show elevated acute phase reactants driven by comorbidities rather than articular inflammation.

Limited prognostic precision: While RF/ACPA positivity indicates higher risk, they incompletely predict individual trajectories, treatment response, or optimal therapeutic intensity.

Non-specificity: RF elevation occurs in other autoimmune diseases, chronic infections, and healthy elderly individuals, limiting diagnostic confidence when present in isolation.

## 4. Novel Autoantibody Systems: Extending the Serological Toolkit

Multiple autoantibody systems targeting post-translationally modified proteins have emerged as potential diagnostic and prognostic biomarkers [3,15]. Although these autoantibodies extend the serological spectrum of RA, most remain outside current classification criteria and should be interpreted as adjunctive rather than definitive diagnostic tools. Table 1 summarizes their characteristics, validation status, and clinical positioning. A unifying theme across these systems is their potential to identify RA patients missed by traditional serology—the seronegative population—representing a key unmet clinical need [4].

### 4.1. Anti-Carbamylated Protein Antibodies (Anti-CarP)

Anti-CarP antibodies recognize proteins modified through homocitrullination, occurring when cyanate binds to lysine residues under inflammatory conditions or smoking exposure [16]. Critically present in 16–30% of ACPA-negative patients (Table 1), anti-CarP provides genuinely additive diagnostic information for the seronegative population [17]. Prospective studies demonstrate that positivity confers a 2–3-fold increased risk of radiographic progression, independent of RF and ACPA status, and these antibodies may precede clinical disease by up to 14 years, suggesting utility in preclinical risk stratification [16,17,18]. Standardized commercial assays are emerging but are not yet widely available.

Clinical positioning: Consider testing in seronegative early arthritis with high clinical suspicion for RA, or in seropositive patients where additional prognostic stratification would influence treatment intensity decisions. This is not yet guideline-endorsed.

### 4.2. Anti-Peptidylarginine Deiminase Antibodies (Anti-PAD)

PAD enzymes catalyze citrullination; antibodies targeting PAD4 and cross-reactive anti-PAD3/4 antibodies (Table 1) associate with aggressive disease phenotypes [19,20]. Anti-PAD3/4 demonstrates particularly strong associations with erosive progression (approximately 3-fold increased risk) and interstitial lung disease [19]. These antibodies stratify severity within the ACPA-positive population rather than identifying additional seronegative patients. Assays remain at the research-only stage, limiting their current clinical applicability despite compelling prognostic data [20].

Clinical positioning: They are potentially valuable for prognostic stratification in ACPA-positive patients, particularly for identifying those at the highest risk of aggressive, erosive disease or extra-articular complications. They are currently awaiting commercial assay development and prospective validation.

### 4.3. Anti-Acetylated Protein and Anti-MAA Antibodies

Antibodies against acetylated proteins (targeting modified vimentin, histones) and malondialdehyde–acetaldehyde adducts represent earlier-stage discoveries [21,22]. While prevalence data suggest potential utility in seronegative disease (15–20% positivity), findings derive from relatively small cohorts with heterogeneous assay methodologies [21]. Anti-MAA antibodies may link RA to cardiovascular risk through shared oxidative stress pathways, but this hypothesis requires prospective validation [22]. Currently, these markers should be considered investigational [15].

Clinical positioning: They are not ready for clinical use. Promising mechanistic links have been found, but with insufficient validation. Results should be awaited from larger, prospective studies with standardized assays before considering implementation.

## 5. Cellular and Molecular Biomarkers

### 5.1. Calprotectin (S100A8/A9)

Calprotectin, a calcium-binding protein released from activated neutrophils and monocytes, has emerged as a sensitive inflammation marker with practical clinical applicability [5,26]. Serum calprotectin levels correlate strongly with disease activity (r = 0.5–0.7 with DAS28) and outperform CRP in detecting subclinical synovitis confirmed by ultrasound or MRI [5]. Elevated calprotectin in patients achieving clinical remission predicts subsequent flare with hazard ratios of 2–3, providing objective data to guide de-escalation decisions [26,27]. Proposed thresholds vary across studies (typically 0.5–1.5 μg/mL for low disease activity), though these are not guideline-endorsed.

Clinical take-home: From a clinical perspective, calprotectin may be useful in selected situations characterized by discordance between clinical assessment and conventional inflammatory markers, or when treatment de-escalation is being considered. However, variability in assay platforms and proposed thresholds currently limits its use as a standalone decision-making tool.

### 5.2. 14-3-3η Protein

The 14-3-3η protein is an intracellular chaperone released during joint damage, reflecting active tissue destruction [23]. Elevated in 60–75% of RA patients with a specificity of 78–93%, this marker demonstrates particularly strong additive value; combined RF, ACPA, and 14-3-3η testing achieves sensitivity exceeding 80% for early RA [23,25]. Critically, 14-3-3η is elevated in 45–58% of seronegative patients, substantially improving detection in this challenging population [25]. Elevated levels predict erosive progression with odds ratios of 2–4, and a commercially available assay (14-3-3η Plus) facilitates clinical adoption [24].

Clinical take-home: Among novel serological biomarkers, 14-3-3η shows the strongest validation data and commercial availability, though it is not yet incorporated into major classification guidelines. It should be considered in seronegative early inflammatory arthritis or seropositive patients with borderline findings. Elevated 14-3-3η identifies higher erosive risk regardless of RF/ACPA status. Commercial assay (14-3-3η Plus) is available.

### 5.3. Cytokines and B Cell Markers: Mechanistic Insights, Limited Clinical Utility

While cytokine profiling (TNF-α, IL-6, IL-17) and B cell phenotyping (plasmablast frequencies, memory B cell subsets) provide valuable mechanistic insights, their translation to routine clinical practice remains limited. IL-6 levels correlate with disease activity but are confounded by on-target effects of IL-6 receptor blockade, which suppresses CRP independently of disease control. Complex flow cytometric B cell analysis requires specialized laboratory infrastructure unavailable in most clinical settings. Single-cytokine measurements lack the specificity and reproducibility required for individual patient decision-making.

Clinical take-home: These markers inform pathophysiology research but are not ready for application in routine care. Complexity and variability preclude their implementation outside of specialized research protocols.

## 6. Genetic and Epigenetic Biomarkers: Future Directions

The HLA-DRB1 shared epitope remains the strongest genetic risk factor for ACPA-positive RA [28]. Genome-wide association studies have identified over 100 additional susceptibility loci, enabling the construction of polygenic risk scores (PRS) [29]. However, current PRS performance (AUC 0.65–0.70) provides insufficient discrimination for individual clinical decision-making [30]. These scores find application primarily in research settings studying at-risk populations (first-degree relatives of RA patients, individuals with undifferentiated arthritis) rather than routine diagnostic workup.

Epigenetic modifications—DNA methylation patterns, histone modifications, circulating microRNAs (particularly miR-146a, miR-155, miR-223)—show altered profiles in RA and correlate with disease activity in research studies [31,32]. However, no epigenetic assay is currently recommended for diagnosis or disease activity monitoring outside clinical trials. Barriers include the lack of standardized platforms, batch effects across laboratories, and poor reproducibility of findings across independent cohorts [32].

Clinical positioning: They should currently be considered as investigational tools for research and at-risk population stratification, not routine clinical biomarkers. Implementation awaits their improved predictive performance and demonstration of actionable utility.

## 7. Multi-Biomarker Disease Activity Assessment

The multi-biomarker disease activity (MBDA) score (Vectra DA) represents the most clinically developed composite biomarker approach, measuring 12 serum proteins across inflammatory, destructive, and metabolic pathways [33]. The algorithm-derived score (1–100 scale) categorizes disease activity as low (<30), moderate (30–44), or high (>44), correlating with DAS28-CRP (r ≈ 0.5) and predicting radiographic progression independent of clinical assessment [34,35]. High MBDA scores confer an approximately 3-fold increased risk of radiographic progression compared to low scores, providing objective risk stratification beyond clinical measures alone [35].

Clinical positioning and controversies: MBDA should be viewed as a complementary risk-stratification tool, not a replacement for clinical assessment. Its most relevant applications include (1) patients in apparent clinical remission where subclinical inflammation is suspected; (2) discordant findings between clinical scores and symptoms; (3) research settings requiring objective, reproducible disease activity measurement [33,36]. Importantly, MBDA-guided treatment has not yet demonstrated superiority over standard treat-to-target strategies in randomized outcome trials—a critical evidence gap for guideline incorporation [12]. Additional limitations constrain broader adoption: performance varies across treatment contexts (particularly with IL-6 inhibitors and JAK inhibitors that directly affect measured analytes) [36], obesity and comorbid inflammatory conditions may confound results, cost limits accessibility, and MBDA is notably absent from major EULAR treat-to-target recommendations [12]. Current evidence supports targeted application in selected clinical scenarios rather than routine use for all RA patients. Importantly, the absence of MBDA from current EULAR recommendations reflects the lack of outcome-driven trials rather than a dismissal of its biological relevance

## 8. Predictive Biomarkers for Treatment Response: An Unmet Need

While this review has focused extensively on diagnostic, prognostic, and disease activity biomarkers, a critical gap remains: the absence of robust, validated biomarkers predicting response to specific therapeutic mechanisms [37,38]. Despite the proliferation of targeted therapies—TNF inhibitors, IL-6 receptor blockers, JAK inhibitors, B cell-depleting agents, T-cell co-stimulation modulators—treatment selection remains largely empiric, guided by comorbidities, safety profiles, and patient preference rather than biomarker-based mechanism matching [39]. This represents perhaps the most significant unmet need in RA biomarker research.

Why predictive biomarkers remain elusive: Pathway redundancy means that blocking one mechanism may lead to its compensation by others. Treatment effects confound interpretation—IL-6 inhibitors suppress CRP independent of disease control. Heterogeneity exists even within seropositive RA, and most studies are retrospective and underpowered [36,40].

Exploratory signals warranting further investigation: Despite these challenges, several preliminary findings merit mention without overselling their clinical readiness. Baseline type I interferon gene signatures have shown associations with differential response to TNF inhibitors versus other mechanisms in some cohorts, though replication has been inconsistent [37]. Synovial pathotypes, as discussed below, offer perhaps the strongest signal—B cell-rich synovitis predicts rituximab response while pauci-immune/fibroid pathotypes predict poor response to multiple biologics [41,42]. Autoantibody characteristics beyond simple positivity—including ACPA fine specificity, epitope spreading, and titer dynamics—may carry predictive information, with some data suggesting that high-titer ACPA patients respond preferentially to abatacept [39]. Serum cytokine ratios and soluble receptor levels have been explored, but lack consistency across studies.

Clinical implications: At present, no predictive biomarker can reliably guide selection between available targeted therapies in routine clinical practice [12]. The R4RA trial’s synovial pathotyping approach (detailed in Section 10) represents the most advanced attempt at biomarker-stratified treatment selection but remains at a research-only stage [41,43]. Until validated predictive biomarkers emerge, treatment decisions will continue to rely on clinical factors, with biomarkers contributing primarily to prognostic stratification and disease activity monitoring rather than mechanism-specific prediction.

## 9. Imaging-Based Biomarkers

Imaging modalities function as both diagnostic/monitoring tools and biomarkers of disease activity and structural damage risk [44]. Their roles differ; musculoskeletal ultrasound offers accessibility and real-time assessment suitable for routine practice, while MRI provides superior sensitivity for early changes but requires greater resources.

### 9.1. Musculoskeletal Ultrasound

Power Doppler ultrasound quantifies synovial vascularity as a marker of active inflammation, with OMERACT-EULAR standardized scoring enabling reproducible assessment [45]. Ultrasound-detected subclinical synovitis in patients achieving clinical remission predicts flare (relative risk 2–3) and radiographic progression, establishing clear prognostic value [6]. Point-of-care ultrasound is being increasingly integrated into rheumatology practice, enabling immediate clinical decision-making at relatively low cost [44].

Clinical take-home: Ultrasound represents an accessible imaging biomarker appropriate for routine use [44,45]. It should be considered for assessing disease activity when clinical findings are equivocal, evaluating remission quality before treatment de-escalation, and monitoring response in patients with persistent symptoms despite normal laboratory markers.

### 9.2. Magnetic Resonance Imaging

MRI offers superior sensitivity for detecting early synovitis, tenosynovitis, bone marrow edema (BME), and erosions before radiographic visibility [46]. BME strongly predicts subsequent erosive progression (odds ratio 4–6), establishing it as a key prognostic biomarker and the single most powerful imaging predictor of structural damage [46,47]. The RAMRIS (Rheumatoid Arthritis MRI Scoring) system provides standardized quantification of synovitis, BME, and erosions across multiple joints [48]. ‘Imaging remission’, incorporating the absence of synovitis and BME, has been proposed as a treat-to-target goal with superior structural outcomes compared to clinical remission alone. Some clinical trials have incorporated MRI endpoints, demonstrating that achieving imaging remission is associated with sustained drug-free remission and reduced flare rates [47]. However, MRI is not yet widely implemented as a routine monitoring tool due to cost, accessibility, time requirements, and absence from current treat-to-target recommendations [12]. Dynamic contrast-enhanced MRI and emerging quantitative techniques may eventually enable more precise inflammation assessment, though standardization remains challenging [48].

Clinical take-home: MRI should be reserved for specific clinical questions, e.g., early disease where diagnosis remains uncertain despite clinical and serological evaluation, baseline prognostic assessment in early RA to identify high-risk patients, or research protocols targeting imaging remission. Routine serial MRI monitoring is not currently justified outside trials.

## 10. Metabolomic and Proteomic Approaches

High-throughput technologies enable comprehensive molecular profiling, with metabolomic studies identifying altered amino acid metabolism, lipid profiles, and oxidative stress signatures distinguishing RA from healthy controls [49]. Emerging evidence suggests that metabolic signatures may predict treatment response; for example, baseline branched-chain amino acid and tryptophan pathway metabolites have shown preliminary associations with TNF inhibitor response in small cohorts. Similarly, proteomic analyses of serum and synovial fluid have nominated numerous candidate biomarkers correlating with disease activity and outcomes [11].

However, critical barriers impede clinical translation: lack of standardized platforms across laboratories, significant batch effects requiring careful harmonization, high dimensionality creating risk of overfitting and false discovery, and poor reproducibility of findings across independent cohorts [11,49]. No metabolomic or proteomic signature currently meets validation standards for clinical implementation.

Clinical positioning: They are firmly in the discovery phase, though there is the potential for future treatment response prediction and disease subtype identification. Clinical application awaits extensive validation and standardization.

## 11. Integrating Biomarker Modalities: Toward Precision Rheumatology

The future of RA biomarkers lies not in identifying single superior markers but in strategic integration across modalities [1,11]. Combined serological, cellular, and imaging assessment provides complementary information: autoantibodies inform diagnosis and long-term prognosis, inflammatory markers (calprotectin, MBDA) reflect current disease activity, and imaging captures both inflammation and structural damage risk [33,44]. Longitudinal biomarker monitoring rather than single time-point testing enables dynamic risk stratification responsive to treatment effects and disease evolution [12]. This multimodal approach mirrors the biological complexity of RA, where pathogenic mechanisms span autoantibody-driven processes, inflammatory cascades, and tissue-destructive pathways that may operate semi-independently across patients and disease phases [1,3].

### 11.1. Synovial Tissue Biomarkers and Precision Treatment Selection

Synovial tissue analysis represents the most direct window into joint pathobiology and has emerged as a powerful tool for patient stratification [50,51]. Transcriptomic profiling of synovial biopsies has identified distinct pathotypes: lymphoid-myeloid (characterized by B cell aggregates and ectopic lymphoid structures), diffuse-myeloid (macrophage-dominated inflammation), and pauci-immune/fibroid (minimal inflammatory infiltrate with stromal predominance) [52,53]. These pathotypes demonstrate differential treatment responses—a finding with profound implications for precision medicine [54].

The landmark R4RA (Rituximab versus Tocilizumab in Anti-TNF Inadequate Responder) trial provided proof-of-concept for synovial biomarker-guided therapy [41]. Patients with B cell-poor synovial signatures showed significantly inferior responses to rituximab compared to tocilizumab, while B cell-rich pathotypes responded well to B cell depletion [41,43]. This trial demonstrated that tissue-based stratification can identify patients unlikely to benefit from specific mechanisms, potentially sparing them ineffective treatment cycles. Ultrasound-guided synovial biopsy is now feasible in routine rheumatology practice with acceptable safety profiles, removing a historical barrier to tissue-based stratification [51].

The precision medicine vision for RA extends beyond tissue pathotyping to integration of multi-omic data—genomics, transcriptomics, proteomics, and metabolomics—into composite algorithms enabling ‘right drug, right patient, right time’ treatment selection [52,53]. Current research explores whether circulating biomarker signatures can serve as surrogates for synovial pathotypes, potentially enabling non-invasive stratification [42]. While synovial biopsy-based approaches remain largely research tools, their translation to clinical practice is accelerating, with several ongoing trials testing biomarker-stratified treatment algorithms [43].

### 11.2. Integrated Algorithms and Machine Learning Approaches

A conceptually important distinction separates risk stratification biomarkers—which predict outcomes but do not directly guide immediate action (anti-CarP, anti-PAD3/4, MRI bone marrow edema) [16,19,46]—from decision-support biomarkers—which inform specific clinical decisions at the point of care (calprotectin for de-escalation, ultrasound for remission verification, MBDA for discordant cases) [5,6,33]. Conflating these categories leads to confusion about what biomarkers can and cannot deliver; predicting risk is not equivalent to guiding action.

Lessons from failed biomarker approaches: Many promising biomarkers failed clinical translation—gene-expression classifiers did not generalize, multiplex cytokine panels showed poor reproducibility [11,37,49]. These failures inform current standards; independent validation is mandatory, and clinical utility—not merely statistical association—must precede implementation

Machine learning approaches integrating high-dimensional biomarker data show promise for identifying patient subgroups with distinct disease biology and treatment response patterns [53]. The concept of biomarker-defined ‘endotypes’ may ultimately enable the rational matching of patients to therapeutic mechanisms. Early studies applying unsupervised clustering to synovial tissue transcriptomics have identified distinct molecular subtypes with differential responses to targeted therapies, providing proof-of-concept for precision stratification [52,54]. The integration of clinical, serological, imaging, and genetic data into composite algorithms—whether rule-based or machine learning-derived—offers the most promising path to clinically actionable biomarker implementation [11].

**Figure 2:**
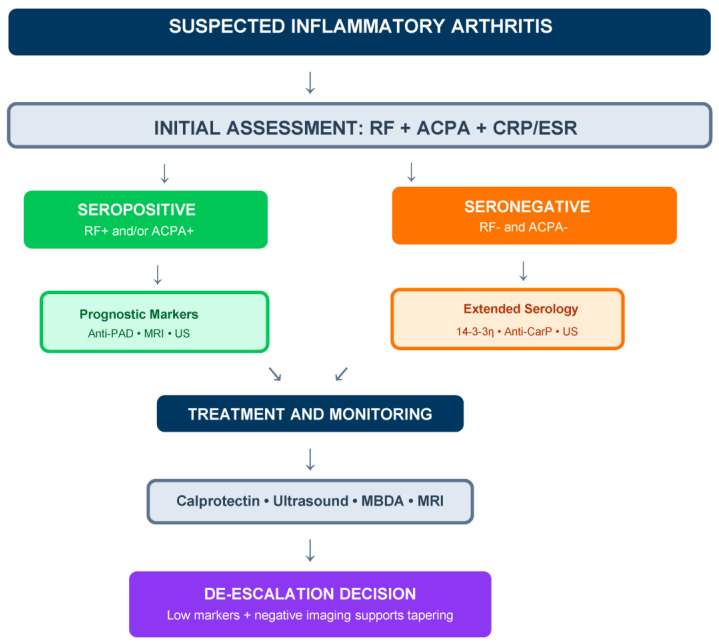
Proposed integrated biomarker algorithm for RA diagnosis and monitoring, illustrating potential incorporation of novel biomarkers into clinical decision-making, intended to support clinical reasoning rather than prescribe mandatory pathways. Biomarkers should support rather than replace clinical judgment and established classification criteria. The algorithm distinguishes approaches for seropositive (prognostic stratification) and seronegative (diagnostic confirmation) scenarios, with longitudinal monitoring and de-escalation guidance. RF: rheumatoid factor; ACPA: anti-citrullinated protein antibodies; Anti-PAD: anti-peptidylarginine deiminase antibodies; Anti-CarP: anti-carbamylated protein antibodies; US: ultrasound; MRI: magnetic resonance imaging; MBDA: multi-biomarker disease activity.

Figure 1 illustrates the hierarchical organization of biomarker categories, while Figure 2 proposes an integrated algorithm incorporating traditional and novel markers into clinical decision-making. These frameworks are conceptual and illustrative; novel biomarkers should support rather than replace clinical judgment and established classification criteria [2,12].

## 12. Clinical Implementation: Prerequisites and Barriers

Translation requires assay harmonization, external validation, cost-effectiveness demonstration, and integration into clinical workflows (Table 2).

The translation of biomarkers into routine clinical practice requires several prerequisites, including assay harmonization across laboratories, external validation in independent and diverse populations, demonstration of cost-effectiveness, regulatory approval where applicable, integration into clinical workflows and electronic health records, and appropriate clinician education. Critically, the absence of most novel biomarkers from current international guidelines reflects the lack of outcome-driven randomized trials rather than insufficient biological validity. Economic and access considerations further constrain implementation; advanced biomarker testing, including multi-biomarker scores, imaging, and multi-omic platforms, entails substantial costs, variable reimbursement, and unequal availability across healthcare systems, raising concerns about equity of access. Moreover, commonly used inflammatory biomarkers capture biological activity and structural risk but incompletely reflect patient-reported outcomes, such as pain, fatigue, and functional impairment, which may persist independently of measurable inflammation due to central sensitization or comorbidities. These limitations underscore that biomarkers should support—but not replace—longitudinal, holistic clinical assessment, and that future progress will depend on integrated, validated, and clinically actionable strategies demonstrating clear benefit over standard care.

Biomarkers for extra-articular manifestations and systemic complications: While this review has focused primarily on biomarkers for joint disease activity and radiographic progression, RA is increasingly recognized as a systemic disease with significant extra-articular manifestations (EAMs), including interstitial lung disease (ILD), cardiovascular complications, and venous thromboembolism (VTE). The biomarker landscape for these systemic complications remains substantially less developed than that for articular disease. Anti-MDA5 antibodies and KL-6 have shown promise for ILD risk stratification, while traditional cardiovascular risk markers remain inadequate for capturing the excess cardiovascular burden attributable to RA-specific inflammation. Real-life evidence has demonstrated increased thrombotic risk in RA patients; for example, Conforti et al. reported a higher incidence of VTE in a real-life RA cohort, supporting the clinical relevance of persistent systemic inflammation and endothelial dysfunction in driving residual thrombotic risk [55]. Dedicated biomarker development for these systemic phenotypes represents an important area for future investigation.

Inflammaging and thrombotic risk as unmet biomarker needs: The concept of inflammaging—chronic, low-grade inflammation associated with aging—intersects importantly with RA pathophysiology and represents a significant unmet need from a biomarker perspective. The challenge is not the absence of inflammatory markers, but rather that commonly used markers such as CRP, IL-6-related markers, and calprotectin are shared across multiple conditions and cannot reliably distinguish active RA inflammation from age-related low-grade inflammation or pro-thrombotic states. This overlap creates difficulty in identifying patients who remain at elevated cardiovascular or thrombotic risk despite achieving clinical remission. Post-infectious models of chronic inflammation may provide relevant insights; Conforti et al. demonstrated that pro-inflammatory cytokines serve as early predictors of chronic rheumatologic disease following Chikungunya virus infection, illustrating how persistent inflammatory signals can drive long-term immune dysregulation and inflammaging beyond joint involvement [56]. Potential solutions may emerge from integrated approaches combining inflammatory markers with markers of endothelial dysfunction and coagulation activation, supported by longitudinal real-life data. Such multimodal strategies could help define inflammaging and thrombotic risk as systemic phenotypes distinct from joint disease activity, though substantial validation work remains before clinical implementation.

## 13. Conclusions and Recommendations

The expanding RA biomarker landscape reflects substantial advances in disease biology, yet translation into routine clinical practice remains uneven. Many biomarkers demonstrate biological plausibility and reproducible associations but lack the outcome-based evidence required for guideline incorporation. Precision rheumatology is therefore best viewed as an incremental process rather than an imminent paradigm shift. Based on current evidence, we offer the following recommendations for clinical practice and research priorities.

For clinical practice: Among novel serological markers, 14-3-3η protein offers the strongest validation and commercial availability for seronegative early arthritis triage [23,24,25]; its addition to RF and ACPA testing improves diagnostic sensitivity meaningfully without substantial loss of specificity, though it remains absent from current ACR/EULAR classification criteria. Calprotectin provides a practical tool for detecting subclinical inflammation when clinical-laboratory discordance is suspected, or treatment de-escalation is contemplated, and elevated levels despite clinical remission should prompt caution regarding therapy reduction [5,26,27]; however, calprotectin cut-offs are not yet standardized across assays and await formal guideline endorsement. Musculoskeletal ultrasound should be integrated into routine practice as an accessible imaging biomarker for disease activity assessment and remission verification, particularly valuable for point-of-care decision-making [44,45]. MBDA testing may benefit selected patients with discordant findings, though limitations described constrain broader adoption [33,34,35,36].

For research priorities: Anti-CarP and anti-PAD antibody assay standardization should be prioritized to enable clinical translation of these promising prognostic markers [16,17,18,19,20]. Prospective randomized trials comparing biomarker-guided treatment strategies to standard care are essential for demonstrating clinical utility and achieving guideline incorporation—observational associations, however strong, are insufficient for changing practice [12]. The development of integrated algorithms combining serological, imaging, and clinical data—potentially leveraging machine learning—represents a promising direction toward precision rheumatology [52,53]. Health economic analyses must accompany biomarker development to ensure cost-effectiveness and inform reimbursement decisions.

Achieving this vision requires sustained investment in validation studies, standardization efforts, implementation science, and equitable access strategies ensuring that advances benefit all patients regardless of geographic or socioeconomic circumstances. The biomarkers reviewed herein represent substantial progress toward these goals—yet the defining advance in RA management will come not from discovering another biomarker, but from learning to use existing ones intelligently, together, and over time.

## Figures and Tables

**Table 1:** Novel autoantibody biomarkers: characteristics, validation status, and clinical utility.

Biomarker	Prevalence	Specificity	In SNRA	Validation	Additive?	Key Clinical Utility	Refs.
Anti-CarP	39–45%	89–96%	16–30%	Validated	Yes	2–3× progression risk; preclinical detection	[16,17,18]
Anti-PAD4	25–45%	92–98%	8–15%	Validated	Partial	Aggressive phenotype; EAMs	[19,20]
Anti-PAD3/4	18–25%	>95%	5–10%	Research	In ACPA+	3× erosive risk; ILD association	[19,20]
Anti-acetylated	35–45%	85–92%	15–20%	Discovery	Unclear	Emerging; heterogeneous assays	[21]
Anti-MAA	30–40%	80–88%	12–18%	Discovery	Unclear	CV risk linkage; small cohorts	[22]
14-3-3η protein	60–75%	78–93%	45–58%	Near-clinical	Yes, strong	Erosive prediction; commercial assay	[23,24,25]

Abbreviations: SNRA—seronegative rheumatoid arthritis (RF−/ACPA−); EAMs—extra-articular manifestations; ILD—interstitial lung disease; CV—cardiovascular; ACPA—anti-citrullinated protein antibodies; Anti-CarP—anti-carbamylated protein antibodies; Anti-PAD—anti-peptidylarginine deiminase antibodies; Anti-MAA—anti-malondialdehyde–acetaldehyde antibodies. Notes: Prevalence and specificity ranges reflect variability across cohorts and assay platforms. “Additive” indicates whether the biomarker provides diagnostic/prognostic value beyond RF and ACPA. Validation stages: Validated = replicated in multiple independent cohorts; Research = promising but requires further validation; Near-clinical = commercially available with substantial clinical evidence; Discovery = early-stage investigation. Approaching clinical readiness (commercial assay available, prospective validation data, though not yet guideline-endorsed).

**Table 2:** Clinical readiness assessment of emerging RA biomarkers.

Biomarker	Evidence	Availability	Guidelines	Best Use Case	Key Barrier	Refs.
Calprotectin	Strong	Commercial	Not endorsed	Remission; de-escalation	Threshold variability	[5,26,27]
14-3-3η	Strong	Commercial	Not endorsed	Seronegative early RA	Cost; awareness	[23,24,25]
Anti-CarP	Moderate	Emerging	Not endorsed	SNRA; prognosis	Assay standardization	[16,17,18]
MBDA (Vectra)	Strong	Commercial	Not endorsed	Discordant remission	Cost; Rx effects	[33,34,35,36]
US Power Doppler	Strong	Widespread	Acknowledged	Activity; remission	Training; scoring	[6,44,45]
MRI BME/synovitis	Strong	Limited	Acknowledged	Baseline prognosis	Cost; access	[46,47,48]
Genetic/Epigenetic	Emerging	Research	Not endorsed	At-risk populations	Validation needed	[28,29,30,31,32]

Abbreviations: RA—rheumatoid arthritis; SNRA—seronegative rheumatoid arthritis; MBDA—multi-biomarker disease activity; US—ultrasound; MRI—magnetic resonance imaging; BME—bone marrow edema; Anti-CarP—anti-carbamylated protein antibodies; Rx—treatment/medication. Evidence strength: Strong = multiple prospective studies and/or meta-analyses; Moderate = replicated in independent cohorts; Emerging = preliminary data requiring validation. Guideline status: “Acknowledged” indicates mention in EULAR/ACR recommendations or imaging guidelines; “Not endorsed” indicates absence from current guideline recommendations despite available evidence. Color coding: Evidence column—green (strong), yellow (moderate), amber (emerging). Guidelines column—green (acknowledged), red (not endorsed).

## Data Availability

No new data were created or analyzed in this study. Data sharing is not applicable to this article.

## Abbreviations

ACPA—anti-citrullinated protein antibodies; ACR—American College of Rheumatology; Anti-CarP—anti-carbamylated protein antibodies; Anti-MAA—anti-malondialdehyde–acetaldehyde antibodies; Anti-PAD—anti-peptidylarginine deiminase antibodies; AUC—area under the curve; BME—bone marrow edema; CDAI—Clinical Disease Activity Index; CRP—C-reactive protein; CV—cardiovascular; DAS28—Disease Activity Score 28-joint count; DMARD—disease-modifying antirheumatic drug; EAMs—extra-articular manifestations; ESR—erythrocyte sedimentation rate; EULAR—European League Against Rheumatism; HLA—human leukocyte antigen; ILD—interstitial lung disease; IL—interleukin; JAK—Janus kinase; MBDA—multi-biomarker disease activity; MHC—major histocompatibility complex; miRNA—microRNA; MRI—magnetic resonance imaging; OMERACT—Outcome Measures in Rheumatology; PRS—polygenic risk score; RA—rheumatoid arthritis; RAMRIS—Rheumatoid Arthritis MRI Scoring; RF—rheumatoid factor; SDAI—Simplified Disease Activity Index; SNRA—seronegative rheumatoid arthritis; TNF—tumor necrosis factor; US—ultrasound; VTE—venous thromboembolism.
